# Annotations

**Published:** 1890-11-08

**Authors:** 


### Annotations.

**Facts about Tubercle.**

Medical men, like other classes of persons, are apt to be led away in large numbers by hypotheses and notions started in high quarters. Times without number have nearly the whole body of practitioners in this and other countries given unquestioning adherence to theories of disease and methods of cure which have been found to have neither consonance with reason nor basis in fact. The present is a critical moment in the history of tubercle and tubercular phthisis, and the treatment of the various diseases described or partly described by these terms. The most urgent need of the moment in medical circles is that of a critical faculty. There is only one thing more discreditable than the acceptance of conclusions on insufficient data by scientific men, and that is the giving of verdicts and the pronouncing of sentences by judges when the evidence has not been sufficient for conviction. Certain indisputable facts are known about tubercle, and medical men should marshal those facts before the eyes of their imagination at the present moment, and with those before them they should resolve to accept nothing new unless it be proved by unquestionable demonstration. It is well known, for example, that even during so recent a period as the last twenty years there have not only been twenty, but far more than twenty, different views about the nature and origin of tubercle, some of them having been flatly contradictory of each other. The opposing opinions have been held by men of equal eminence and scientific authority in different countries. In this connection ic is sufficient to mention such names as Virchow, Wilson Fox, Burdon Sanderson, Cohnheim, Villemin, Andrew Clark, Klebs, Aufrecht, Toussaint, and Koch. Whilst it has long been known that inoculation by a great variety of putrid and non-putrid materials would produce tubercle, it has never been asserted until within the last year or two that tubercle always springs from a definite germ, the tubercle bacillus, and can never spring from anything else. Of course it is conceivable that the small plant now called by the name of tubercle bacillus may have its habitat in twenty different substances, some of them so far apart as a diseased lymphatic gland and a piece of simple white thread. It is conceivable, we say, but not at all likely. But if it be true that tubercle can only arise from the tubercle bacillus, then we have to accept the hypothesis that this bacillus is practically ubiquitous, and that fifty things, or for that matter five hundred, which have hitherto been considered quite harmless may be the abode of the deadly vermin. This may be the fact; but we ask for its proofs.

**Voluntary Patients in Lunatic Asylums.**

It has been the practice in Scotland for a considerable number of years to allow persons to become patients in lunatic asylums without demanding the usual certificate of lunacy either from themselves or their friends. But can a single person be found either in Scotland or elsewhere who will voluntarily consign himself to a lunatic asylum? A good many such persons have been found in sober Scotland during the past ten years, and last year no less than seventy-six separate entries took place. Seventy-six persons, whose mental faculties were impaired, had yet so much saving common sense left that they voluntarily surrendered themselves to the restraints of an asylum with the object of cure. This speaks volumes for the excellence of the management of Scotch asylums on the one hand, and for the rational freedom from prejudice of the Scottish people on the other. A few months ago a medical friend of the writer's reported his own experience as a voluntary patient in a well-known Scotch asylum. His mental aberration, of which he had himself been painfully conscious before entering the institution, had been entirely cured by a twelvemonths' residence there. Several years ago the writer saw a lady at another Scotch asylum, who was there for a second voluntary term of treatment. She stated to an assembled group of practitioners and students that some years before she had been so insane as to attempt the life of one of her own children. She was then certified as a lunatic, and compulsorily moved to the asylum. After being there about a year she completely recovered, and was allowed to return home. She remained quite well for two or three years; but under the mental strain of increasing family worries, she felt the old feelings of insanity coming back again, and had the good sense to ask to be removed to the asylum before she reached a stage at which she could no longer control herself. This time she recovered in a few months, and once more returned home. She remained well for four or five years, almost twice as long as before. Then she became conscious of the beginnings of her old troubles. Resolved to nip the mischief in the bud, she resorted at once to the friendly shelter of the asylum and the kind but firm management of the intelligent superintendent. In six weeks her health was restored, and she was ready to go home again. It was the day before her departure that the writer, with other medical men and students, heard her story from her own lips. The moral of these facts is very obvious! Every lunatic asylum should cease to be called by that opprobrious name, and should become and be regarded as a hospital for mental diseases. So far as possible, patients who are conscious of a condition of mental worry which they feel almost incapable of controlling, should voluntarily enter such hospitals for a period of treatment. That is the rational way and the only rational way of dealing with the ever-increasing number of mental diseases produced by the stress and pressure of modern life. Lunacy—the name is hateful and absurd—mental aberration as we ought to call it, is an effect of purely physical causes, either inherited or acquired. Even under the certifying system, when patients are allowed to become quite mad before treatment is commenced, cures are numerous, and many of them permanent. But if we could eradicate from the popular mind and memory the evil associations of lunatic asylums, and change those institutions into hospitals for mental diseases, and persuade mentally worried and impulsive people to be treated at the commencement of their troubles, we should diminish the number of our lunatics by at least three-fourths. These considerations demand the attention not only of the Lunacy Commissioners and of the heads of lunatic asylums, but still more of the intelligent public who form the opinions of the people and influence their actions.

**Starvation and Indigestion.**

Dr. James Goodhart writes an amusing letter to the *British Medical Journal*, in which he incidentally touches upon starvation diet as a method of curing indigestion. He supposes the case of "a poor woman (these cases are seldom males), who, being perhaps out of sorts, and a little distended and uncomfortable after her meals, thinks she has 'indigestion,' and leaves off one of the components of a diet already far too meagre. . . No relief is obtained, and another goes by the board, and yet another, and so on till her intakings are so infinitesimal that I am not far from the belief that such people, inasmuch as they still live, must have acquired the art of assimilating the nitrogenous emanations of their own environment. . . I would say that in this large group, the medical adviser may with advantage insist on a gradual return to ordinary diet, and he will often find that the stomach, after a period of recalcitrancy, will submit and consent to digest ordinary food. . . The treatment that these patients want is a short lesson in physiology. They cannot be expected to understand that their stomachs have lost their power from want of use; that the regaining of that power will certainly be attended with some discomfort; and that the method of cure is, least of all, medicine, most of all the gradual increase of the work the stomach has to do." This is extremely sensible so far as it goes, but not perhaps quite correct in its suggestions as to cure. A starvation diet for dyspeptics is seldom or never demanded; on the contrary, what is usually required is a large variety of tempting and digestible food. When Dr. Goodhart says that medicine is the last thing needed, we think he over-states his case in his desire to be epigrammatical and striking. If he had said that the ordinary remedies for dyspepsia in the class of cases mentioned were generally useless, we should have at once agreed; but experience shows that acid and bitter tonics, effervescing preparations of steel, quinine and iron, and the like, will often work wonders when the ordinary dyspepsia remedies entirely fail. Many such cases begin to recover immediately on removal to the seaside or to the bracing atmosphere of the hills. The point to understand, as Dr. Goodhart states, is, that these so-called dyspeptics are only dyspeptics secondarily. Their real and primary trouble is a worried and broken down nervous system. This is to be cured, and with it the dyspepsia, by change of air, nerve tonics, or blood enrichers; or, better still, by all three combined.

